# Medical and psychosocial implications of adolescent extreme obesity – acceptance and effects of structured care, short: Youth with Extreme Obesity Study (YES)

**DOI:** 10.1186/1471-2458-13-789

**Published:** 2013-08-29

**Authors:** Martin Wabitsch, Anja Moss, Thomas Reinehr, Susanna Wiegand, Wieland Kiess, André Scherag, Reinhard Holl, Rolf Holle, Johannes Hebebrand

**Affiliations:** 1 Division of Pediatric Endocrinology and Diabetes, Interdisciplinary Obesity Unit, Department of Pediatrics and Adolescent Medicine, Ulm University, Eythstr. 24, D-89073 Ulm, Germany; 2 Vestische Childrens Hospital, University Witten/Herdecke, Dr. F. Steiner Str. 5, D-45711 Datteln, Germany; 3 Charité Universitätsmedizin Berlin, Ambulantes Adipositas Zentrum, Interdisziplinäres SPZ der Kinderklinik, Augustenburger Platz 1, D-13353 Berlin, Germany; 4 Hospital for Children and Adolescents, Department of Women and Child Health, University Hospitals, University of Leipzig, Liebigstraße 20a, D-04103 Leipzig, Germany; 5 Institute for Medical Informatics, Biometry and Epidemiology and Center for Clinical Trials Essen (ZKSE), University Hospital Essen, University of Duisburg-Essen, Hufelandstr. 55, D-45122 Essen, Germany; 6 Institute for Epidemiology and Medical Biometry, Ulm University, Albert-Einstein-Allee 41, D-89081 Ulm, Germany; 7 Institute of Health Economics and Health Care Management, Helmholtz Zentrum München – German Research Center for Environmental Health, Ingolstädter Landstr. 1, D-87564 Neuherberg, Germany; 8 Department of Child and Adolescent Psychiatry, University of Duisburg-Essen, LVR-Klinikum, Wickenburgstr. 21, D-45147 Essen, Germany

**Keywords:** Adolescents with extreme obesity, Bariatric surgery, Social isolation, School and vocational integration

## Abstract

**Background:**

Prevalence rates of overweight and obesity have increased in German children and adolescents in the last three decades. Adolescents with extreme obesity represent a distinct risk group. On the basis of data obtained by the German Child and Youth Survey (KiGGS) and the German district military offices we estimate that the group of extremely obese adolescents (BMI ≥ 35 kg/m^2^) currently encompasses approximately 200.000 adolescents aged 14 to 21 yrs. Conventional approaches focusing on weight reduction have largely proven futile for them. In addition, only a small percentage of adolescents with extreme obesity seek actively treatment for obesity while contributing disproportionately strong to health care costs. Because of somatic and psychiatric co-morbidities and social problems adolescents with extreme obesity require special attention within the medical care system.

We have initiated the project “Medical and psychosocial implications of adolescents with extreme obesity - acceptance and effects of structured care, short: ‘Youths with Extreme Obesity Study (YES)’”, which aims at improving the medical care and social support structures for youths with extreme obesity in Germany.

**Methods/Design:**

We focus on identification of these subjects (baseline examination) and their acceptance of diagnostic and subsequent treatment procedures. In a randomized controlled trial (RCT) we will investigate the effectiveness of a low key group intervention not focusing on weight loss but aimed at the provision of obesity related information, alleviation of social isolation, school and vocational integration and improvement of self-esteem in comparison to a control group treated in a conventional way with focus on weight loss. Interested individuals who fulfill current recommended criteria for weight loss surgery will be provided with a structured preparation and follow-up programs. All subjects will be monitored within a long-term observational study to elucidate medical and psychosocial outcomes. Our aim is to evaluate realistic treatment options. Therefore inclusion and exclusion criteria are minimized.

We will recruit adolescents (age range 14–21 years) with extreme obesity (BMI ≥ 35 kg/m^2^) (extreme group) within 24 months (120 per centre, 5 centres) as well as obese adolescents being at risk for developing extreme obesity (BMI ≥ 30 – 34.9 kg/m^2^) (at risk group). Follow-up evalutations will be performed biannually after inclusion for several years depending on additional funding. In sum, we aim at establishing evaluated health care structures for extremely obese adolescents.

**Discussion:**

The results of YES will be of importance for a frequently neglected group of individuals, for whom current medicine has little to offer in terms of structured access to empirically evaluated therapeutic programs. Thus, the results will be *both* a help for the adolescents within the study and for others in the future given that the trial will lead to a positive finding. Moreover, it will help practitioners and therapists to deal with this neglected group of individuals.

**Trial registration:**

Project registration numbers for each subproject: 1.) ClinicalTrials.gov: NCT01625325, NCT01703273, NCT01662271, NCT01632098; 2.) Germanctr.de: DRKS00004172, DRKS00004195, DRKS00004198, DRKS00004197.

## Background

The increase in the prevalence rate of obesity in pediatric populations is accompanied by an even more pronounced increase of the rate of extreme obesity [1-3]. The effect of extreme obesity in adolescence is multi-fold, affecting individuals during both adolescence and adulthood, their families, and the health care system. Extreme obesity shows substantial tracking into adulthood and entails elevated mortality and high rates of co-morbid disorders [4,5]. Similarly, adolescents with extreme obesity experience increased mortality and morbidity [6-12] and global impairments in daily functioning [13].

Somatic conditions frequently associated with severe obesity include premature death, heart disease, obstructive sleep apnea, hypertension, dyslipidemia, and type 2 diabetes mellitus, which have significant and well-documented cardiac, renal, and ophthalmic complications for young adults. Obesity, glucose intolerance, and hypertension in childhood and adolescence are strongly associated with increased rates of premature death [14]. Other serious conditions include pseudotumor cerebri, steatohepatitis, slipped capital femoral epiphysis, cholelithiasis, polycystic ovary syndrome, and early severe degenerative joint disease [15]. Psychiatric morbidity is substantially elevated in treatment seeking extremely obese adolescents with mood, anxiety and eating disorders figuring prominently [16].

In population-based samples morbidity is much less elevated [16,17]. However, the more extreme the cutoff that is used for definition of extreme obesity in adolescence, the more difficult it is to ascertain a sufficiently large group of cases [16]. As a consequence there is a paucity of scientific data on longitudinal somatic and psychosocial development of adolescents with extreme obesity.

Several studies have revealed that the outcomes of conventional treatment including behavioural and pharmacological approaches for adolescents with extreme obesity are poor [18]. In extremely obese adults the only treatment that has proven successful is weight loss surgery. Results of weight loss surgery in adults have been summarized recently [19]. Meta-analyses of primarily observational data in adults has demonstrated that surgery is associated with disappearance or at least intermittent improvement of type 2 diabetes, hypertension, dyslipidemia and sleep apnea in 70-86% of cases [20]. It has been reported that weight loss surgery significantly improved psychosocial functioning [21], quality of life [22], and physical function [23]. In general, gastric banding resulted in less weight loss in comparison to bypass operations.

However, there are several possible complications of weight loss surgery. In adults perioperative mortality rates are 0.1-0.5% and immediate postoperative complications occur in 10% of individuals. Diversionary procedures increase the long-term risk of nutrient deficiency (up to 50% of patients) while gastric bands can slip (6% of patients) or erode (10%) necessitating re-operation [24].

In adolescents, weight loss surgery is at an experimental stage. Preliminary data in adolescents suggest that short-term outcomes for weight loss and improvement of comorbidities are similar to those observed in adults, but further studies are necessary to confirm these results and evaluate long-term outcomes [18,25-28]. Early evidence of safety and efficacy exists for two procedures (adjustable gastric band, Roux-en-Y gastric bypass (RYGB)) [15,29-31]. In principle, the group of adolescent patients with extreme obesity may be particularly vulnerable to long-term complications of surgery; on the other hand the group could potentially maximally benefit via for example enhanced integration into the job market and reduction of social isolation.

Although weight loss surgery is still at an experimental stage in adolescents there is currently a significant increase in adolescents being operated [32]. This is also true in Germany [Lennerz BS, Wabitsch M, Lippert H, Wolff S, Knoll C, Weiner R, Manger T, Kiess W, Stroh C: Bariatric Surgery in Adolescents and Young Adults – Safety and Effectiveness in a Cohort of 345 Patients, submitted]. However, neither structured preparation nor post-treatment follow-up programs currently exist for these patients.

Given the above mentioned challenges, we have identified “Medical and psychosocial implications of adolescent extreme obesity – acceptance and effects of structured care” as an important research issue. We have developed a recruitment approach for adolescents with extreme obesity involving various institutions to which these adolescents have contact to as well as our well established clinical treatment programs and two German registries (APV, CrescNet). We have chosen a synergistic and coordinated activity involving four regional networks (Leipzig (East) [33], Essen/Datteln (West), Berlin (North), Ulm (South)) which include the five most experienced University treatment centres for obese adolescents in Germany.

## Methods/Design and research aims

**Figure 1:**
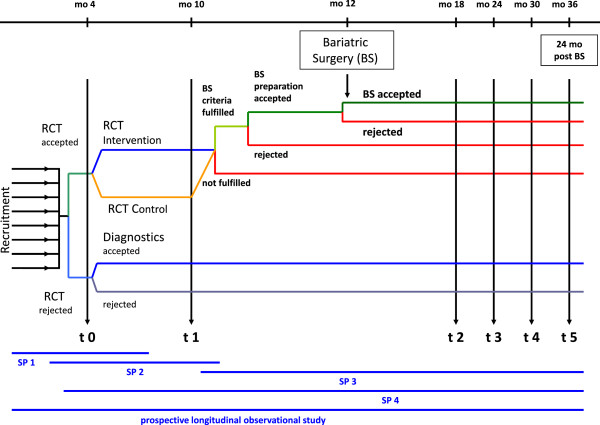
**Overall design of the entire research project ‘Youth with Extreme Obesity Study’ (YES).** This figure gives an overview about the successive built up of the subprojects 1 to 4 as well as the longitudinal observational cohort study within YES. SP1, 2, 3, 4 = subproject 1, 2, 3, 4; RCT = randomized controlled trial; BS = bariatric surgery; mo = month/s; t0, 1, 2, 3, 4, 5 = timepoints for baseline and follow-up examinations.

The project “Medical and psychosocial implications of adolescent extreme obesity - acceptance and effects of structured care, short: ‘Youths with Extreme obesity Study (YES)’” consists of four subprojects and a longitudinal observational cohort study which are successively built on each other. An overview about the overall design of the entire research project is given in Figure 1.

This multicenter study will be conducted in accordance to the Helsinki Declaration and the ICH-GCP guideline, and to other nationally valid regulations.

The project protocols describing the research involving human subjects and the collection of human material and human data were proofed by the ethical committees of the 5 study centers (Ulm University, Charité Berlin, University of Leipzig, University Duisburg-Essen, and University Witten/Herdecke).

### Subproject 1

In subproject 1 “Identification and assessment of adolescents with extreme obesity” we identify extremely obese adolescents in different settings with the aim of providing them with an assessment to allow diagnosis of relevant somatic disorders; we use questionnaires to dimensionally assess depressiveness and other psychopathological traits. Since only a small percentage actively seeks treatment for weight reduction, we have developed a recruitment approach for adolescents with extreme obesity involving various institutions to which these adolescents have contact to as well as our well established treatment programs and two German registries (APV, CrescNet).

This subproject addresses the following research questions:

1. What percentage of subjects is willing to accept a diagnostic work up in relationship to the respective ascertainment setting?

2. What are the predictors for the acceptance of the diagnostic work-up?

3. What are the prevalence rates and how severe are obesity related medical and mental comorbidities?

4. What percentage of subjects is interested in a treatment of the diagnosed comorbidities?

5. What factors predict this interest?

6. How can the living conditions, the psychosocial situation and the health-related quality of life be characterised in adolescents with extreme obesity?

7. What percentage of adolescents is willing to attend the low level intervention outlined in subproject (SP) 2?

8. What factors predict this willingness?

### Methods for recruitment of patients in subproject 1

We will screen adolescents aged 14 to 21 years with extreme obesity (BMI ≥ 35 kg/m^2^) in SP 1 over a 24 months period. We will additionally recruit a group of sex and age matched obese adolescents at risk for extreme obesity (BMI: 30.0 – 34.9 kg/m^2^). The obtained results will be compared between the two obesity groups. The results will help to understand the barriers which restrain adolescents with extreme obesity from actively seeking diagnostic and therapeutic health services. This knowledge will improve future health care services for these patients.

**Figure 2:**
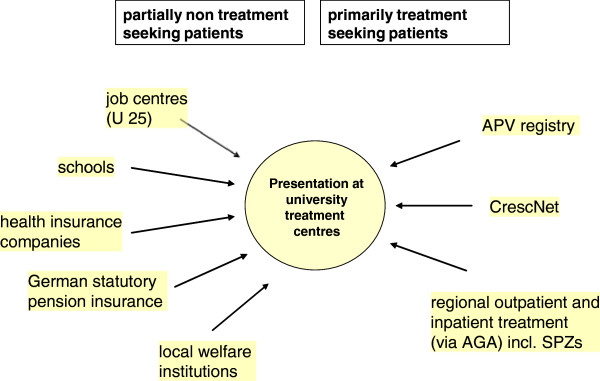
**Recruitment strategies.** This figure shows the different recruitment strategies (partially non-treatment seeking and primarily treatment seeking) for the ‘Youth with Extreme Obesity Study’ (YES).

The different recruitment strategies are shown in Figure 2.

Although no population-representative sample can be recruited, we have taken all possible measures to motivate patients with extreme obesity in the four regions to join the study and to consider the diagnostic and medical care provided. Each individual potentially eligible receives ample information. This will be supported by regular announcements by the local mass media.

### Methods for baseline examination in subproject 1

Baseline assessments include an array of standardized questionnaires and validated instruments to assess health, psycho-social situation, dimensional psychopathology and health related quality of life, as well as an in-depth medical evaluation. The somatic, psychiatric, and socio-demographic evaluation of the patients will be performed according to the Germany guidelines for adolescents with extreme obesity (http://www.a-g-a.de) and the protocol of the Teen-LABS study (http://www.cincinnatichildrens.org/research/project/teen-labs/default.htm). We will use protocols of The German Health Interview and Examination Survey for Children and Adolescents (KiGGS-protocols) (http://www.kiggs.de/service/english/index.html) to assess social status, eating and physical activity behaviour.

### Successive built up of subprojects 1, 2, 3 and 4 as well as the longitudinal observational cohort study

The identification and cross-sectional assessment of adolescents with extreme obesity in subproject 1 of YES will form the basis for subproject 2 (Randomized controlled manual-based low-level intervention) and subproject 3 (Structured preparation, pre- and post-surgery treatment of adolescents with extreme obesity) of YES. All patients assessed in SP 1, followed up in SPs 2 and 3 will constitute a well characterized cohort of adolescents with extreme obesity. This cohort will be prospectively followed-up with examinations every six months in a longitudinal observational cohort study as part of YES that will cover a maximum of nine years (maximal duration of grant). Thereby, we will gain insight into the course of adolescent extreme obesity and the development of the medical and psychosocial comorbidities during adolescence and young adulthood. We will perform subgroup analyses based on the treatment options these youths have chosen. We will determine the percentage of patients who can be integrated into the job market or into an apprenticeship training position. Finally, in subproject 4 “Economic aspects of extreme obesity in adolescents” of YES we will analyze the economic burden of extreme obesity in adolescents.

### Subproject 2

In SP 2 “A Structured, manual-based low-level intervention vs. treatment as usual Evaluated in a randomized controlled trial for adolescents with extreme obesity – the STEREO trial” of YES we propose a new innovative low level intervention to overcome treatment abstaining behaviour. The new intervention will not focus on weight loss. We will evaluate efficacy and safety of the new intervention in comparison to treatment as usual. The RCT will be conducted according to “good clinical practice” and is professionally supported by the Center for Clinical Trials Essen.

The new innovative intervention will be a manual based low key group intervention with 6 sessions over the course of 3–6 months focusing on the improvement of quality of life and psychosocial functioning. The topics covered are: social competency, body image, coping with weight related stigmatization, victimization and bullying, coping with stress, life satisfaction, and psychological well being. The intervention does not primarily focus on weight loss.

The active comparator (“treatment as usual”; TAU) is a manual based group intervention as proposed by the treatment guidelines in Germany for routine obesity care. The 6 sessions over the course of 3–6 months focus on weight loss. The topics covered are causes, consequences and treatment options of obesity, nutrition, eating behaviours and problem solving strategies, self esteem and emotional eating, physical activity and exercise, media usage.

Based on the current state of knowledge, we will perform a confirmatory test of two *a priori* hierarchically ordered hypotheses controlling the family-wise significance level α (two-sided) at 5% [34]. The primary research questions are:

1. The compliance rate 6 months after randomization differs between subjects in the new low key intervention group compared to the TAU group.

2. Covariant-adjusted changes in health-related quality of life (HRQoL) (assessed by the DISABKIDS scale; Chronic Generic Module) between baseline and the 6 months follow-up will be different in the low key intervention group compared to the TAU group.

The sample size calculation was driven by the second of the two research questions reflecting empirical evidence on standardized effect sizes from the literature (e.g. [35] and the presumed smaller effect size. However, detailled power considerations also demonstrated that the power will be larger than 80% if the compliance rates differ by more than 15% points for a range of compliance rates under TAU (continuity corrected Chi^2^ test). In terms of null (H_0_) and alternative (H_1_) hypotheses for the second research question we will test: H_0_: Δ_NEW_ = Δ_TAU_ against H_1_: Δ_NEW_#Δ_TAU_ where Δ. is the covariant-adjusted expected (i.e., mean) change in HRQoL between baseline and the 6 months follow-up in the respective group. Two independent groups with 146 adolescents each have a power of 80% to detect mean differences of 0.33 in units of standardized effect sizes (Cohen’s d; *t*-test with equal variances; α = 0.05 (two-sided)). Allowing for ~15% “Drop-outs” we decided to ascertain a total 2 × 175 = 350 adolescents for SP 2 and will screen a total of 1,200 adolescents for the complete YES project based on expectations regarding the participation in subprojects.

Secondary outcome measures include a responder analysis (combining compliance rate and changes in HRQoL), changes in the 6 subscales of DISABKIDS, changes in quality of life (KIDSCREEN-52), changes in self-esteem (Rosenberg’s scale patient questionnaire), changes in self reported time spent outside the home (questionnaire), changes in depression symptoms (Becks Depression Inventory 2), changes in perceived stress (Fliege scale questionnaire), changes in self reported attendance of school, apprenticeship, or work as well as changes in self reported physician and therapist contacts.

Individuals who complete the baseline evaluations in SP 1 will be invited to participate in 6 group sessions over a 6 months period. Group assignment to the low key intervention vs. standard care group will be at random. The effects of the interventions on health related quality of life and psycho-social functioning will be assessed by questionnaires 6 months after the treatment start. Subsequently, subjects will be invited to participate in SP 3 and the longitudinal follow-up examinations of YES.

### Subproject 3

The aim of the SP 3 “Safety and effectiveness of weight loss surgery in adolescents with extreme obesity within a structured pre- and post-surgery treatment programme – Observational study” of YES is to generate observational data of safety and effectiveness of weight loss surgery in adolescents with extreme obesity.

Adolescents with extreme obesity identified and assessed in SPs 1 and 2, who 1) fulfil the formal criteria for bariatric surgery [15,32,36,37], 2) have shown compliance with either one of the intervention programs (SP 2) and 3) are willing to consider weight loss surgery, will participate in a two month long manual based preoperative information program to ensure that the adolescents know potential risks and benefits. They will then be able to give informed consent (as well as their parents in case of minors). Such adolescents will undergo weight loss surgery in one of the five treatment centers which have all established collaborations with bariatric surgery units. After surgery, the patients will participate in a manual-based post-operative treatment programme. 7From an ethical viewpoint, we will have taken extensive precautions to make sure that the adolescents can give truly informed consent. Because the adolescents will have to complete *both* the low level intervention program outlined in SP 2 and the 2 month long presurgical program, they will be selected for maximal compliance which we consider as the major predictor for a good outcome of bariatric surgery.

We will use standardized techniques and measures to assess the short, medium and long term results of bariatric surgery. We will compare post-surgical outcomes to pre-operative status and examine risks and benefits of surgery. The study will be performed on the basis of the protocols of the US-Teen-LABS study [38]. As a control group we will examine adolescents with extreme obesity who successfully completed SP 2 and who either declined participation in the pre-surgery program outlined in this proposal or declined surgery after completion of the pre-surgery program using propensity-score methods. A further comparison group is formed by those subjects who take part in SP1 but refuse to participate in the RCT (SP2) if they are available for follow-up investigations.

The following objectives will be assessed: 

1. identification of those extremely obese adolescents best suited for bariatric surgery,

2. developing a structured patient program to be completed prior to and after surgery,

3. use the observational data to generate (potentially biased) effect sizes estimates of safety and effectiveness.

Finally, we will also determine associations between clinical/demographic patient characteristics, components of the surgical procedure with post-operative risks and changes in patient status.

The primary endpoint of SP3 is weight change defined as difference between BMI at date of surgery and 12 months after. This outcome choice is in accordance with the outcome of the Teen-LABS study. The other secondary outcome measures are change of BMI between date of surgery and after 24 months, changes (date of surgery - 12 months) in somatic and psychosocial co-morbidities of obesity as described in SP 1, and changes (date of surgery - 12 months) in percentage of self-reported probands´ attendance of school, vocational training or work (see SP2).

Perioperative and postoperative safety (adverse and serious adverse events) will be investigated using a standardized protocol [39] including risks of bariatric surgery, nutrient deficiencies, and adherence to nutritional supplements.

There will be different types of bariatric procedures performed in adolescents with extreme obesity. The course of treatment (preoperative, postoperative, follow-up) as well as the impact on quality of life has so far hardly been investigated. We will therefore develop a disease- and treatment-specific QoL-instrument.

Our study will result in follow-up data for at least three different surgery techniques allowing us to descriptively compare the results and possibly to establish criteria for the method of choice for a specific patient in the future or to stimulate the conductance of a head-to-head comparison in an RCT setting. Moreover, we will compare the results obtained in this study with those obtained in the US- Teen-LABS study [39].

### Subproject 4

In subproject 4 “Economic aspects of extreme obesity in adolescents” of YES we will analyze the economic burden of extreme obesity in adolescents. This research project will investigate the impact of extreme obesity on healthcare utilization, costs and health related quality of life in a cohort of adolescents. An additional focus is the economic evaluation of a bariatric surgery intervention for adolescents with extreme obesity.

The first aim of this subproject is to measure, value and analyze all relevant direct and indirect costs, to describe differences between BMI categories among obese adolescents as well as first results on pre-post interventional differences.

We hypothesize that cost induced by utilization of healthcare services of extremely obese (BMI ≥ 35 kg/m^2^) adolescents will be significantly higher compared to the group with a BMI between 30 and 35 kg/m^2^ and, in the subgroup with bariatric surgery, will decrease after the intervention.

Cost measurement will primarily focus on health care costs induced by utilization of healthcare services and on productivity losses.

The basis of utilization and cost analyses is the cross sectional sample of obese (30 < BMI < 35 kg/m^2^) and extremely obese (BMI ≥ 35 kg/m^2^) adolescents who will serve as the basis for the cohort and the embedded clinical studies. Data on utilization of healthcare services and production losses, if adolescents or their parents were unable to attend work due to illness of the adolescent, will be retrospectively assessed over the previous 6 months via the use of standardized question- naires. In detail, the following variables will be included: number and frequency of physician visits, number of admissions to hospitals and rehabilitation services, medication, further types of care such as physical therapy and alternative medicine and work absenteeism. Utilization data and work absenteeism will be monetarily valued [40] and adapted to the year of the studies. The results will be compared first, between BMI categories and second, between the groups with and without surgical intervention.

Additional cost data will be collected every six months after the intervention (within the clinical follow-up) to enable a pre- post comparison. Further analyses of possible economic implications in the long-run (e.g. skill attainment, academic performance, future labour market outcomes, risk of unemployment etc.) (e.g. [41,42]) will be performed and methods for their economic assessment will be explored based on additional research of the existing literature.

Intervention costs will be calculated using diagnosis related group cost rates.

Second, we aim to measure HRQoL to find differ- ences between BMI categories among obese children as well as identify other factors influencing HRQoL.

We hypothesize that extremely obese (BMI ≥ 35 kg/m^2^) adolescents have significantly lower HRQoL compared to the group with a BMI between 30 and 35 and that, in subgroups with specific interventions (low level intervention in SP2, bariatric surgery SP3), HRQoL will increase after the intervention.

In order to calculate QALYs, the German version of the EQ-5D questionnaire (five items) including the visual analogue scale (EQ-VAS) will be used as a generic instrument for which a preference- based index exists. Measurements will take place at baseline and every six months after the intervention (within the clinical follow-up). Hennessy and Kind reported adequate performance of the EQ-5D questionnaire in adolescents [43].

The third aim of SP4 is to examine the cost-effectiveness of bariatric surgery in extremely obese adolescents compared to the group without bariatric surgery from a societal perspective based on first post-surgical results (short term) and to prepare cost-effectiveness-analyses based on follow-up data (medium term). Further analyses of possible long term consequences (e.g. adverse health effects, future labour market outcomes etc.) will be performed based on literature.

We hypothesize that the costs of the intervention will be accompanied by strong positive effects such as relative weight loss and QALYs gained and therefore the intervention will show good results regarding cost-effectiveness. We further hypothesize that the high costs of bariatric surgery will be amortized by health savings due to less utilization of health care services and lower indirect costs in the medium to long term (third funding phase).

In future research phases, we intend to collect follow-up data with respect to utilization, costs and quality of life for comprehensive pre-post comparisons and cost-effectiveness analyses. We expect to provide valid and up-to-date information on costs and health-related quality of life for this relevant patient group. This information will be of value for planning future preventive and therapeutic strategies within the German health care system.

### Longitudinal observational cohort study

All adolescents who participated in the baseline examination (SP1) of YES are to be recruited for a longitudinal observational cohort study that will cover a minimum of nine years. We will gain insight into the natural course of adolescent extreme obesity; we will also perform subgroup analyses based on the treatment options that we originally provided these youths with. We will determine the percentage of patients who can be integrated into the job market or into an apprenticeship training position.

## Discussion

The project “Medical and psychosocial implications of adolescent extreme obesity - acceptance and effects of structured care, short: ‘Youth with Extreme obesity Study (YES)’”, is a research project which aims at improving the medical care and social support structures for adolescents with extreme obesity in Germany. The overall goals of the project are to 1) provide comprehensible information on a) obesity, nutrition and physical activity, b) current treatment options, c) the need for regular medical contacts to diagnose and treat co-morbid disorders at an early stage, 2) increase self-esteem, 3) enable re-initiation of social contacts and 4) provide help with respect to educational and vocational needs. These overall goals will be achieved within the 4 subprojects and a longitudinal observational cohort study.

The specific goals are 1) to identify extremely obese adolescents in different settings with the aim of providing them with a diagnostic assessment including relevant somatic and psychiatric disorders (subproject 1), 2) to study the safety and efficacy of an innovative low level intervention to overcome the treatment abstaining behaviour (subproject 2), 3) to provide observational study estimates on measures of safety and effectiveness of weight loss surgery in adolescents with extreme obesity (subproject 3), 4) to investigate the impact of extreme obesity on healthcare utilization, costs and health related quality of life (subproject 4) and 5) to establish a longitudinal observational cohort study for adolescents with extreme obesity in order to gain insight into the natural course of adolescent extreme obesity. We will also determine the percentage of patients who can be integrated into the job market or into an apprenticeship training position.

Extremely obese adolescents have a strongly elevated risk for early death, somatic comorbidities, psychiatric disorders, and social isolation including unemployment due to both functional impairment and stigmatisation. Despite the dire implications of adolescent extreme obesity and the frequent overt (e.g. orthopaedic disorders) and non-overt (e.g. hypertension) comorbidities, these adolescents are difficult to reach and treat in medical terms. In fact, only a small percentage of individuals actively seek treatment. The underlying reasons are poorly understood and presumably include the young age, a predominantly low educational and socioeconomic status, and functional impairment due to inactivity and psychiatric co-morbidities. Unsuccessful attempts to lose weight by themselfes and/or within the medical system may have led to frustration with respect to treatment seeking behaviour.

In acknowledgement of these difficulties, we have developed a recruitment approach for adolescents with extreme obesity involving various institutions to which these adolescents have contact to as well as our well established treatment programs and two German registries (APV, CrescNet). We have chosen a synergistic and coordinated activity involving four regional networks (Leipzig (East), Essen/Datteln (West), Berlin (North), Ulm (South)) which include the five most experienced University treatment centres for obese adolescents in Germany.

Successful treatment of extreme adolescent obesity is currently realistically not possible using non-surgical approaches. Instead of attempting to evaluate another treatment regimen aimed at weight loss and/or caloric restriction and/or increased energy expenditure, we perceive the need to shift the focus to other important needs of this high risk group. With an innovative intervention in SP2 of YES we want to overcome the therapeutic nihilism frequently associated with adolescent obesity by evaluating a low level manual based intervention in combination with access to a small local network of specialists. This approach focuses on improving the psychosocial functioning in contrast to a weight loss focus. The proposed programme will be the first multisite RCT for the comparison of a manual-based low level intervention with a focus on psychosocial functioning vs. treatment as usual (with its focus on weight loss) in extremely obese adolescents.

If our intervention is successful, we aim to provide institutions such as school systems, job centers and insurance companies with a low key and comparatively inexpensive intervention program for which enhanced compliance and increased psychosocial functioning has been demonstrated. The minimal inclusion requirements to join the intervention and the fact that intervention is manual-based will enhance dissemination. We believe that it is important that the obese adolescent receives all the information required to realistically come to terms with his/her condition and to get a perspective on the important health issues related to obesity. In addition, the intervention will address the importance of motivation and personal behaviour to reduce/surmount social isolation, psychological malfunctioning, and to increase the awareness for continuous treatment of comorbid disorders. Finally, we perceive the RCT as an important element of pre-surgery evaluation of extremely obese adolescents. It will help to structure the access to this invasive therapy.

The only treatment that has proven successful in terms of weight loss for extremely obese adults is weight loss surgery, which is at an experimental stage only for adolescents. Due to both the lethality and the high long-term risks associated with weight loss surgery physicians are generally reluctant to recommend this treatment option at young ages. However, in the light of the serious somatic, psychiatric and psychosocial implications of morbid obesity, we propose here to systematically assess this therapeutic venue in SP3 “Safety and effectiveness of weight loss surgery in adolescents with extreme obesity within a structured pre- and post-surgery treatment programme – Observational study”.

The conclusions based on this study are limited by the general restrictions of an observational study and we view our study as a starting point for to examine the risks and benefits of surgery in adolescents.

This part of the project is needed now because we face an increase in the number of young bariatric surgery patients in Germany [Lennerz BS, Wabitsch M, Lippert H, Wolff S, Knoll C, Weiner R, Manger T, Kiess W, Stroh C: Bariatric Surgery in Adolescents and Young Adults – Safety and Effectiveness in a Cohort of 345 Patients, submitted]. The concern about the haphazard application of these procedures in adolescents, and the lack of sufficient long-term data on safety and efficacy of weight loss surgery at these young ages as well as the lack of a quality management for the pre- and post-operative treatment clearly demonstrate the need for a study to systematically assess the efficacy, safety, and health care costs of these procedures in adolescents using an observational study design [32,44].

This part of YES (subproject 3) is the first prospective, multicenter clinical observation study on weight loss surgery in adolescents with extreme obesity in Germany. It is the first study not conducted by a bariatric surgeon. It will also include structured pre- and post-operative treatment programs applying the same manual and examination procedures at each of the centers. The study will result in observational data to estimate for short and longer term safety and efficacy of bariatric surgery by comparing post-surgical outcomes to pre-operative status and to the control group of patients not operated and examining risks and benefits of surgery (other adolescents ascertained within this consortium).

On the basis of the results of this study new recommendations (including the possible necessity for well-designed RCTs) on the applicability of weight loss surgery in adolescents with extreme obesity will be established.

Analyzing obesity-associated health care costs (SP4 of YES) can contribute important information to illuminate the societal burden posed by this public health problem, but does not inform on the value of interventions and programs aimed at diminishing or solving the problem. There is a substantial amount of studies evaluating those interventions and programs targeted at children in terms of their effectiveness (usually by looking at changes in height/weight or skin-fold measures) (for a review see e.g. [45]. In contrast to evaluations focussing on effectiveness, the evidence on the cost-effectiveness of such interventions and programs is much more limited. For the majority of interventions proposed or implemented cost-effectiveness information is yet missing. This holds, in particular, for Germany, where, to our knowledge, not one economic evaluation study of an intervention – conventional or bariatric – for extreme obese adolescents has been published until now. This is a significant gap as cost-effectiveness is specific to the health system of study.

In summary, the results of YES will be of importance for a frequently neglected group of individuals, for whom current medicine has little to offer in terms of structured access to empirically evaluated therapeutic programs. Thus, the results will be *both* a help for the adolescents within the study and for others in the future given that the trial will lead to a positive finding. Moreover, it will help practitioners and therapists to deal with this neglected group of individuals.

## Competing interests

The authors declare that they have no competing interests.

## Authors’ contributions

MW and JH have made substantial contribution to study conception and design and wrote the first draft of the manuscript. AS made substantial contribution to study conception and the biometrical design of SP1-3. AM participated in the coordination of the study, wrote sections of the manuscript, created figures and formatted the manuscript. All authors revised sections of the manuscript. All authors read and approved the final manuscript.

## Pre-publication history

The pre-publication history for this paper can be accessed here:

http://www.biomedcentral.com/1471-2458/13/789/prepub
